# Isothermal diagnostic assays for the detection of soil-transmitted helminths based on the SmartAmp2 method

**DOI:** 10.1186/s13071-017-2420-1

**Published:** 2017-10-18

**Authors:** Nour Rashwan, Aïssatou Diawara, Marilyn E. Scott, Roger K. Prichard

**Affiliations:** 1 Institute of Parasitology and Centre for Host-Parasite Interactions, Macdonald College, McGill University, Ste Anne de Bellevue, QC, Canada; 2 Department of Medical Parasitology, Faculty of Medicine, Al-Azhar University, Cairo, Egypt; 3 Biology Program, Division of Science and Mathematics, New York University Abu Dhabi, Abu Dhabi, United Arab Emirates

**Keywords:** Soil-transmitted helminths, SmartAmp2, Lamp, Isothermal assay, β-tubulin gene, Species-specific

## Abstract

**Background:**

Diagnosis of soil-transmitted helminths (STHs) has traditionally relied on stool microscopy, which has a number of critical deficiencies. Molecular diagnostics are powerful tools to identify closely related species, but the requirement for costly equipment makes their implementation difficult in low-resource or field settings. Rapid, sensitive and cost-effective diagnostic tools are crucial for accurate estimation of STH infection intensity in MDA programmes in which the goal is to reduce morbidity following repeated rounds of chemotherapy.

**Results:**

In this study, colourimetric isothermal assays were developed using SmartAmp2 primer sets and reagents in loop-mediated amplification (LAMP) assays. Species-specific primer sets, designed on a specific target sequence in the β-tubulin gene, were used to identify *Necator americanus*, *Trichuris trichiura* and *Ascaris lumbricoide*s. After initial optimization on control plasmids and genomic DNA from adult worms, assays were evaluated on field samples. Assays showed high sensitivity and demonstrated high tolerance to inhibitors in spiked faecal samples. Rapid and sensitive colourimetric assays were successfully developed to identify the STHs in field samples using hydroxy napthol blue (HNB) dye.

**Conclusions:**

Rapid and simple colourimetric diagnostic assays, using the SmartAmp2 method, were developed, with the potential to be applied in the field for detection of STH infections and the estimation of response to treatment. However, further validation on large numbers of field samples is needed.

**Electronic supplementary material:**

The online version of this article (10.1186/s13071-017-2420-1) contains supplementary material, which is available to authorized users.

## Background

Soil-transmitted helminths (STHs), including *Ascaris lumbricoides*, *Trichuris trichiura*, the hookworms *Ancylostoma duodenale* and *Necator americanus*, and *Strongyloides stercoralis*, are gastrointestinal nematodes causing human morbidity in tropical and subtropical areas of the world [1]. Pre-school and school-age children are most at risk of heavy infection and of developing severe morbidity [2]. Early childhood infections contribute significantly to malnourishment, stunted growth, intellectual retardation and cognitive deficits [1, 3]. Recent estimates indicate that approximately 900 million children are at high risk of acquiring STH infection and are therefore in need of annual preventative treatment [4].

Mass drug administration (MDA) programmes are the major control strategy and target the most important STH, namely *A. lumbricoides*, hookworms and *T. trichiura*, and involve a single oral dose of albendazole (ALB; 400 mg) or mebendazole (MEB; 500 mg) administered periodically [5, 6]. Both of these front-line anthelmintics are benzimidazoles and concern has been expressed as to the possible selection for resistance to both benzimidazoles [7]. MDA programmes have expanded in the past ten years and an estimated 369 million children were treated in 2015; however, drug coverage reached only 44.7% of school-age children, and 51.4% of pre-school-age children in need in endemic countries [8]. With expansion of MDA programmes for STHs, there is a need to monitor infection levels in the programme areas.

Diagnosis is necessary to determine the response to treatment and endpoint for MDA, with continued screening being required to identify settings with ongoing transmission or relapse [9]. The significant progress of MDA programmes in developing countries, leading to low intensity infections, increases the need for accurate diagnosis to sustain the overall benefits and evaluate the impact of these programmes [6]. Although chronic morbidity caused by STH infections can be eliminated by anthelmintic treatment, inaccurate diagnosis may underestimate prevalence and infection intensity and prevent a reliable estimation of the impact of deworming programmes.

Current diagnostic methods have some deficiencies [10]. Traditional Kato Katz (KK) is the most commonly used method, recommended by the World Health Organization (WHO) because it is simple and relatively inexpensive and provides basic information on prevalence and infection intensity for both STH and *Schistosoma* species [11]. Parasitological techniques are sufficiently sensitive if appropriate sampling methodologies are used; however, their performance is suboptimal in low intensity infections [10], which can result in false estimates of infection intensity [12, 13]. Furthermore, if stool samples are not examined soon after collection, hookworm eggs develop into larvae [14], leading to false negative results. Serology-based assays for detection of antibody or antigen have been developed for a small number of intestinal parasites but their performance is variable and their cost often prevents their implementation in resource-limited settings [15, 16].

Polymerase chain reaction (PCR)-based methodologies have several advantages over existing parasitological and serological methods: they are more sensitive and allow for the detection of a wide variety of pathogens in addition to STHs [17–20]. Additionally, they allow the detection of parasite eggs or larvae even after samples have been stored or frozen [21]. Conventional PCR and real-time PCR (RT-PCR) have achieved critical advances in the detection of several parasitic infections. Several studies have also applied PCR-based assays for sensitive and specific detection of STH DNA in human faecal samples [22, 23].

Quantitative PCR (qPCR)-based assays are accurate, highly sensitive and specific for the diagnosis of STH infections compared to traditional microscopy-based parasite diagnosis [17–19]. They allow the detection of infections in very low-intensity settings. Although PCR-based technologies provide reliable, specific and sensitive tools, they are not widely used in low-income and limited resource-settings as the high costs of reagents, the requirement of expensive equipment, and highly skilled personnel limits their use as a routine diagnostic method [20]. Development of rapid, sensitive and cost-effective methods for the detection of STH infections using molecular diagnostic tools that could be adapted to field conditions is desirable.

Loop-mediated isothermal amplification (LAMP) assays [24], are unique technologies that have emerged as promising approaches for detection and quantification of viral, fungal, bacterial, and parasitic infections. LAMP is a one-step DNA amplification method that amplifies a target sequence under isothermal conditions with high specificity and sensitivity [24, 25]. It uses a strand-displacing DNA polymerase, allowing auto-cycling amplification that leads to accumulation of a large amount of target DNA. The colourimetric detection of DNA amplification enables visual inspection of the results without requiring sophisticated and expensive equipment. Only a heat block or incubator/water bath is required [25]. As the reaction progresses, the by-product magnesium pyrophosphate accumulates. This causes reaction turbidity that can be monitored visually using a variety of metal indicators such as calcein or hydroxy naphthol blue (HNB) [26–28], or the presence of double-stranded DNA that can be measured using intercalating dyes such as SYBR green I. Fourteen neglected tropical diseases (NTD) recognized by the WHO, have been assessed using LAMP assays [27], including schistosomiasis [29], filariasis [30], strongyloidosis [31, 32], and STHs [33, 34]. Compared with other PCR methods, LAMP has unique advantages for field use, mainly in terms of rapidity, simplicity and flexibility in readout. SmartAmp2 (Smart Amplification process) is a specific type of LAMP with unique asymmetrical primer design that makes the assay highly specific under isothermal conditions [35].

The aim of this study was to develop a rapid and accurate diagnostic assay based on the SmartAmp2 method for the detection of *N. americanus* (*A. duodenale* samples were not available)*, T. trichiura* and *A. lumbricoides* and to validate their specificity and reliability in field samples. Species-specific primer sets were designed on a specific target sequence in the β-tubulin gene. The target sequences were chosen because they are unique to each species, while being conserved within each of them. For these reasons, conserved regions of a conserved gene were chosen (the β-tubulin sequence is known for all STH species and was suitable) rather than a multi copy sequence, such as a repeat region, which may show variability between different isolates of the same species. This approach is feasible with the unique specificity of the SmartAmp2 approach combined with the outstanding sensitivity of LAMP detection.

## Methods

### Parasite materials and DNA extraction

*Necator americanus* adult worms, larvae and eggs and *A. lumbricoides* adult worms were available from previous studies of DNA-based detection methods [36, 37]. Adult *T. trichiura* DNA was donated by Dr. Nesjum, University of Copenhagen, Denmark. Faecal samples were collected in Haiti and Panama from children, who were naturally infected. Eggs were either isolated from fresh stool samples and preserved in 70% ethanol, or the stool samples were preserved in 70% ethanol for subsequent egg isolation. Eggs were isolated under a dissecting microscope using a 10 μl pipette. Genomic DNA was extracted from eggs as described [38] and adapted to STHs. Ten μl each of proteinase-K (10 μg/ml) (Invitrogen, Life Technologies; Burlington, ON, CA) and β-mercaptoethanol (Sigma-Aldrich, Oakville, ON, CA) were added to 1 ml lysis buffer [KCl (50 mM), Tris-HCl (10 mM), pH 8.3, MgCl_2_ (2.5 mM), 0.45% Nonidet P-40, 0.45% Tween 20 and 0.01% gelatine] just before use. Twenty-five μl of lysis buffer mix was added to previously isolated eggs and then tubes were incubated at 60 **°**C for 2 h. Genomic DNA was extracted from adult *A. lumbricoides* using DNeasy tissue extraction kit® and from adult *N. americanus* using QIAamp DNA mini kit® (Qiagen, Mississauga, ON, CA) per the manufacturer’s protocol.

### Control plasmid constructs

To develop the SmartAmp2 assays, control plasmids were constructed and used as DNA templates for assay optimization. Specific target regions were selected based on the β-tubulin isotype 1 sequence alignment of *N. americanus*, *A. duodenale*, *A. lumbricoides*, *T. trichiura* and *S. stercoralis* (GenBank: EF392851, EF392850, EU814697, AF034219, AY898944) (http://multalin.toulouse.inra.fr/multalin/) (Additional file 1: Figure S1) to select sequences that have sufficient inter-species variation, for species-specific amplification, with no intra-species variation. PCR amplifications of the specific target regions were performed using the primers shown in Table 1. The PCR master mix contained 2 μl 10× PCR buffer, 1 μl MgSO_4_ (50 mM), 1 μl dNTP (10 mM), 1 μl forward and reverse primers (10 μM) (Invitrogen), 1 U Platinum *Taq* DNA polymerase High Fidelity (Invitrogen), 2 μl genomic DNA and distilled H_2_O (dH_2_O) to reach a final volume of 20 μl. Negative controls (dH_2_O) were also included for quality control. The PCR reaction conditions were 94 °C for 3 min, followed by 35 cycles at 94 °C for 45 s, then 57–59 °C for 45 s, then 68 °C for 1 min, and a final extension at 68 °C for 10 min. The resulting PCR fragments were Sanger sequenced to confirm the presence of the target region. The amplified fragments were cloned into TOPO-TA-Cloning vector (Invitrogen). Plasmid DNAs were extracted and purified using QIAprep Spin Miniprep kit® (Qiagen) and subsequently sequenced by Sanger sequencing at the McGill University/Genome Quebec Innovation Centre, Montreal, Quebec. Purity and quantity of DNA in clones were measured using a Nano Drop photometer (Implen, Munich, Germany). Then control plasmids were used as DNA templates for assay optimization and development.

**Table 1:** Primers for the control plasmid constructs of β-tubulin isotype 1 gene sequences

STH	Primer sequence (5′–3′)
*A. lumbricoides*	Forward: CACATACGGAGACCTCAACC
	Reverse: CAGTAAGGTAACGACCGTGC
*N. americanus*	Forward: CGAACCCAACATATGGAGATC
	Reverse: CCATCATGTTCTTTGCGTCG
*T. trichiura*	Forward: CAACACCAACTTACGGAGAC
	Reverse: GTGACGAGGATCACAAGCAG

### SmartAmp2 primer design

**Fig. 1:**
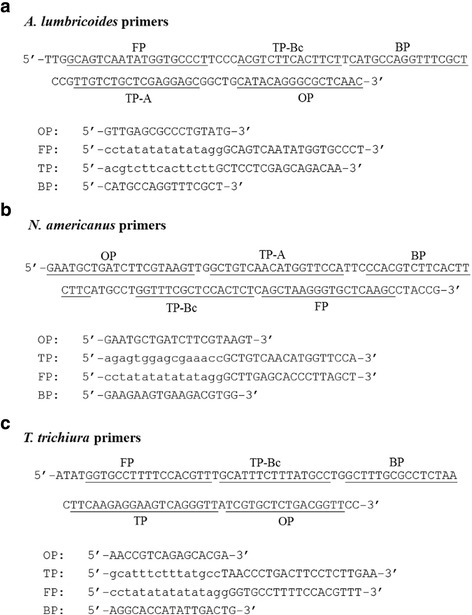
SmartAmp2 primer design. Specific target regions of the β-tubulin isotype 1 gene as well as the sequences of primers for *A. lumbricoides* (**a**), *N. americanus* (**b**) and *T. trichiura* (**c**) assay. *Abbreviations*: OP, outer primer; TP, TP-A/TP-Bc, turn-back; FP, folding primer; BP, boost primer

Selection and optimization of SmartAmp2 primers is important for the accurate detection of a specific DNA target. Sequence alignments were performed using Multalin software http://multalin.toulouse.inra.fr/multalin/ and sequence variations between species were used to design species-specific primers. SmartAmp2 primer sets were designed specifically to amplify and detect a specific target region, in a different exon from those that contain the resistance associated SNPs at codon 167, 198 and 200, in the β-tubulin isotype 1 gene of *A. lumbricoides*, *N. americanus* and *T. trichiura*. At the same time they were designed not to amplify *A. duodenale* or *S. stercoralis*, based on the alignment (Additional file 1: Figure S1). Primers were designed manually and with the guidance of the online software version 1.1 (SMAPDNA), made available by KK.DNAFORM, Japan (http://www.dnaform.jp/en/). Further refinements, in the primer design, were made and the best candidate primer sets were selected after being tested on control plasmids. A set of four specific primers, comprising an outer primer (OP), folding primer (FP), turn-back primer (TP), and boost primer (BP), were designed to recognize five different sequences in the β-tubulin sequence (Fig. 1). All SmartAmp2 primers were regular primers, not HPLC purified (Invitrogen).

### SmartAmp2 assay development and optimization

To optimize the assays, control plasmids were used to develop each assay and to evaluate the sensitivity and specificity between different primer sets. Assays were optimized using different concentrations of betaine, primers, and MgSO_4_. Two *Bst* DNA polymerases, *Bst* 2.0 DNA polymerase and wild-type *Bst* DNA polymerase Large Fragment were evaluated. The SmartAmp2 assays were standardized for optimal reagent concentrations, temperature, and time conditions and carried out in 25 μl reactions containing 2 μM TP/FP, 1 μM BP, 0.25 μM OP (Invitrogen), 1.4 mM dNTPs (Invitrogen), 0.8 M betaine (Sigma-Aldrich), 1× isothermal buffer [20 mM Tris-HCl (pH 8.6), 10 mM KCl, 10 mM (NH_4_)_2_SO_4_, 8 mM MgSO_4_, 0.1% Tween 20], 1/100,000 SYBR Green I (Invitrogen), 1.2 μl *Bst* 2.0 DNA polymerase (New England Biolabs, Whitby, ON, CA) [39] and 1 μl of DNA template. Reactions were incubated at 60 °C for 60 min. Initially, the Rotor-Gene Q system (Qiagen) was used to maintain isothermal conditions and to monitor in real-time the change in fluorescence intensity of SYBR Green I during the reaction. Assays were evaluated in terms of amplification (positive) and non-amplification (negative) within 60 min. Negative controls were always included.

### Specificity and sensitivity of SmartAmp2 diagnostic assays

To verify the specificity of each assay for detection of a specific target DNA, SmartAmp2 primers were tested using DNA from non-target STHs. Each primer set was tested against individual species DNA and mixed STH DNA to assess whether DNA from other STH species would affect the amplification of a target DNA sequence. To determine the sensitivity of each assay, 10-fold dilutions were prepared from 10 ng of purified adult worm genomic DNA. SmartAmp2 reactions were carried out as previously described in triplicate and were repeated twice. Reactions were incubated isothermally, at 60 °C for 60 min, in a RT-PCR apparatus, to monitor the change in fluorescence intensity of SYBR Green I. Negative controls were included in all experiments.

### Colourimetric assay for diagnosis of STH infections

Initially, as part of assay development and optimization, SmartAmp2 products were detected in the RT-PCR apparatus and confirmed by 3% gel electrophoresis. SYBR green was used to allow the visual detection of SmartAmp2 products. The amplification results could be visually inspected by adding 1 μl of 1 /10 diluted SYBR Green I to each tube post-reaction. The colour change from orange to yellow green allows the discrimination between negative and positive results. Post-reaction handling of the SmartAmp2 products has a high risk of contamination, a limitation that suggested we should evaluate the hydroxy napthol blue (HNB) dye. The pre-addition of HNB to the reaction mixture eliminated the need for post-amplification handling. SmartAmp2 reactions were carried out in 25 μl and 120 μM HNB (Sigma-Aldrich) was added to the reaction mixture. All reactions were conducted in duplicate and incubated in a heat block at 60 °C for up to 60 min. The assay was terminated by heating at 80 °C for 5 min, inactivating the *Bst* DNA polymerase and precautions were taken to prevent cross-contamination. To confirm product identity, PCR of the SmartAmp2 product using TP-A and FP primers was examined. Additionally, a parallel real-time SmartAmp2 analysis of the same DNA samples was performed to compare and confirm the efficiency and reliability of the colourimetric assay. Negative controls were included in all experiments.

### Validation of STH diagnostic assays on field samples

Validation for field samples was performed with pools of *A. lumbricoides* (*n* = 21), *N. americanus* (*n* = 19), and *T. trichiura* (*n* = 15) samples obtained from Haiti and Panama. Pools of 10 eggs/larvae previously examined under microscopy for each sample were analyzed. Eggs were digested in 25 μl of lysis buffer mix. From this crude lysate, 3 μl were added to each reaction after a DNA heating step at 95 °C for 3 min. SmartAmp2 assays were carried out as described above. Tubes were incubated at 60 °C for 90 min. Positive and negative controls were always included as references in each experiment.

### Assessment of polymerase tolerance to faecal inhibitors

To further evaluate the efficiency of the SmartAmp2 assays and the tolerance of the *Bst* DNA polymerase to inhibitors in faecal samples, faecal samples that were confirmed to be negative for STH eggs were spiked with a known number of eggs or larvae. Approximately 1 g of faeces preserved in 70% ethanol was centrifuged and the faecal pellet was washed three times in phosphate-buffered saline (PBS) and centrifuged to remove any traces of ethanol. PBS was added to the faecal pellet to a final volume of 1 ml. Ten aliquots of 100 μl (100 mg) of this faecal homogenate were transferred to new tubes. Tubes were centrifuged and excess PBS was removed. Faecal aliquots were spiked with *Ascaris* eggs, *Trichuris* eggs (~10 eggs) or *Necator* larvae (~10 larvae). Other faecal samples were not spiked and were used as negative controls. Spiking experiments were run in triplicate and repeated twice.

DNA was extracted after faecal samples were frozen at -80 °C for 30 min. Then 20 μl of buffer A [NaOH (200 mM) + 2% tween-20] was added to each tube. After a 15 min incubation period at 25 °C, tubes were heated at 99 °C for 10 min. Tubes were allowed to cool, and then 20 μl of Buffer B [Tris-HCl (100 mM) and 2 mM EDTA] were added and a second heat shock at 98 °C for 5 min was performed. Finally, samples were centrifuged and the supernatants were transferred to a new PCR tube. SmartAmp2 assays were assessed using 1–10-fold dilutions of the faecal extracts. Faecal extracts were heated at 95 °C for 3 min, cooled on ice and then 1 μl were added to the SmartAmp2 reaction mixture in a total volume of 10 μl as described above. Bovine serum albumin (BSA) was included in the reaction mixture to stabilise the DNA polymerase and to neutralise faecal inhibitors. Assays were evaluated with or without BSA and with different BSA concentrations (0.2–0.6 μg/μl) (Sigma-Aldrich). Positive and negative controls were always included. The reaction mixtures were incubated at 60 °C for 90 min in a RT-PCR.

## Results

### Selection of SmartAmp2 primers

Several primer sets were designed to specifically amplify a target DNA sequence within the β-tubulin gene. Screening of these primer combinations and assay conditions identified an optimal primer set that completed the amplification within 20–30 min from the target genomic DNA (10 ng). Primer sets were assessed based on speed, yield of amplification and efficiency to differentiate a target sequence from a highly related alternative target. All primer sets that showed delayed or cross-amplification with other STH DNAs were omitted. The location and sequences of primers for each STH target are shown in Fig. 1. A set of four primers was designed TP, FP, BP, and OP. The 3′-end of two or three primers were designed to incorporate sequence variation.

**Fig. 2:**
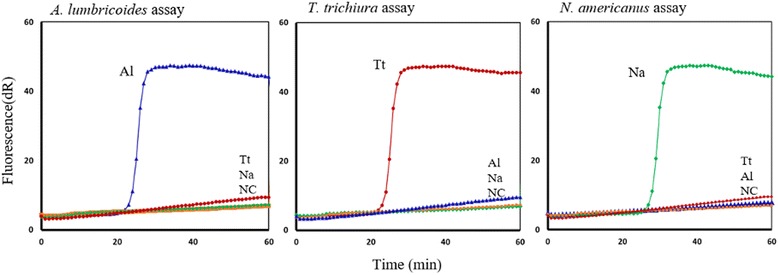
STH SmartAmp2 diagnostic assays. Left, *A. lumbricoides-*specific primers amplified only *A. lumbricoides* DNA (Al) (10 ng). Centre, *T. trichiura-*specific primers amplified *T. trichiura* DNA (Tt) (10 ng). Right, *N. americanus-*specific primers amplified *N. americanus* DNA (Na) (10 ng). Negative controls (NC)

The selected assays specifically amplified the appropriate target genomic DNA; no amplification was detected from non-target STH DNAs. For example, the *A. lumbricoides*-specific primers rapidly amplified *A. lumbricoides* genomic DNA within 20–30 min, whereas the same primers failed to amplify *N. americanus* or *T. trichiura* DNA after 90 min. The same results were achieved for *T. trichiura* and *N. americanus* assays. All experiments were run in duplicate to check the consistency and accuracy of the results. All negative control reactions included in the experiments showed no amplification for up to 90 min (Fig. 2). These results confirmed that the SmartAmp2 assays were optimized, as they accurately differentiated a target DNA sequence from closely related sequences.

### Analytical specificity and sensitivity of STH SmartAmp2 assay

**Fig. 3:**
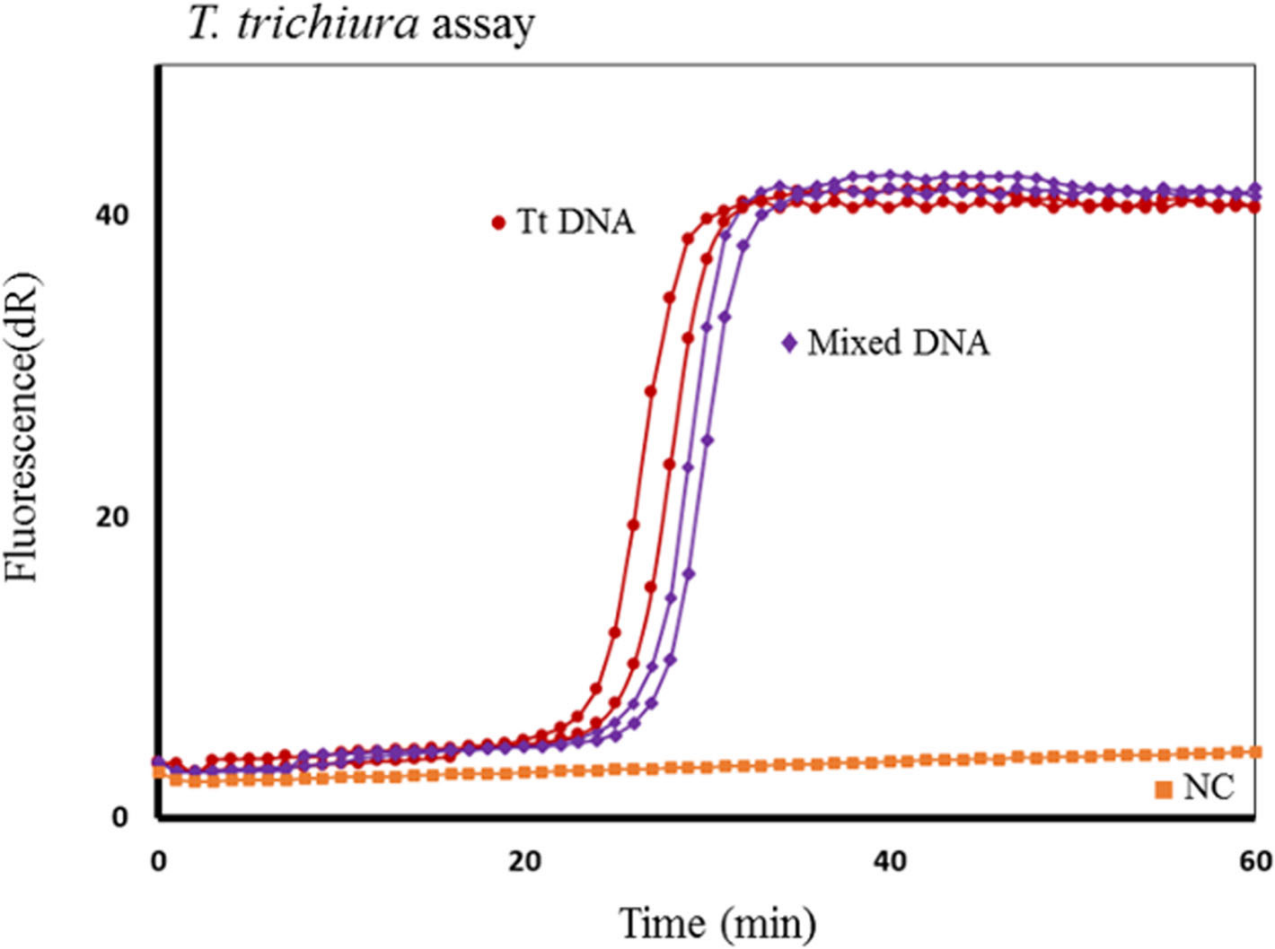
Specificity of STH SmartAmp2 assay. Specificity of the *T. trichiura* diagnostic assay in individual *T*. *trichiura* DNA and mixed STH DNA (T. *trichiura*, *N. americanus* and *A. lumbricoides*). Negative controls (NC)

**Fig. 4:**
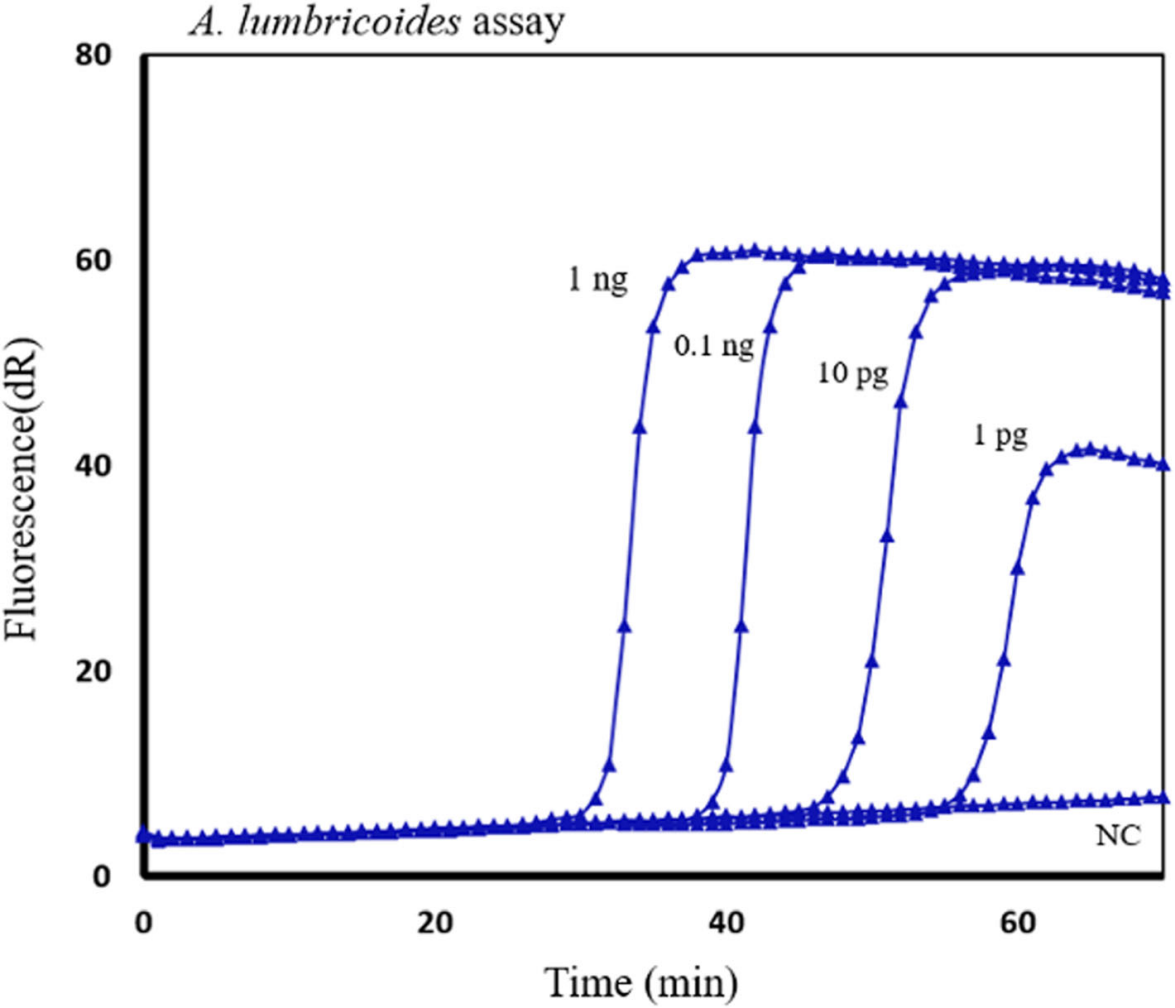
Sensitivity of STH diagnostic assay. SmartAmp2 amplification profile using serial dilutions of *A. lumbricoides* genomic DNA from (1 ng - 1 pg) and negative control (NC) with *A. lumbricoides* primer set as a representative example

Assay specificity was assessed in individual and mixed STH genomic DNA. Each assay was tested in a mix of two or three STH DNAs. The presence of non-target STH DNA even in equal or higher concentrations did not inhibit or affect the specific amplification. The *T. trichiura* primers amplified *T. trichiura* DNA alone or in a mix with *A. lumbricoides* and *N. americanus* DNA with no significant delay or inhibition (Fig. 3). Similar results were also obtained in *A. lumbricoides* and *N. americanus* assays. To assess the analytic sensitivity of the SmartAmp2 assays, the detection limit of DNA was determined by analyzing serial dilutions of genomic DNA. *Ascaris lumbricoides* primers allowed the detection of 1 pg genomic DNA within 50–60 min (Fig. 4). Similar levels of sensitivity were also obtained with the *N. americanus*, and *T. trichiura* primers.

### Colourimetric STH SmartAmp2 assay

**Fig. 5:**
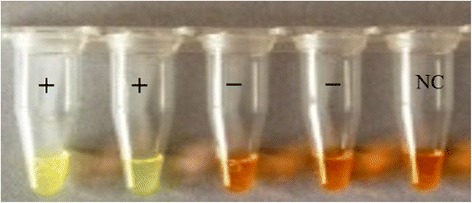
Colourimetric detection of STHs using SYBR Green 1. Colourimetric results of SmartAmp2 assay using *A. lumbricoides* primer set on genomic DNA from *A. lumbricoides* (**+**), *N. americanus* (**−**), *T. trichiura* (**−**) and negative control (NC). The post-addition of SYBR Green to reactions changed from orange (negative) to yellow-green (positive)

**Fig. 6:**
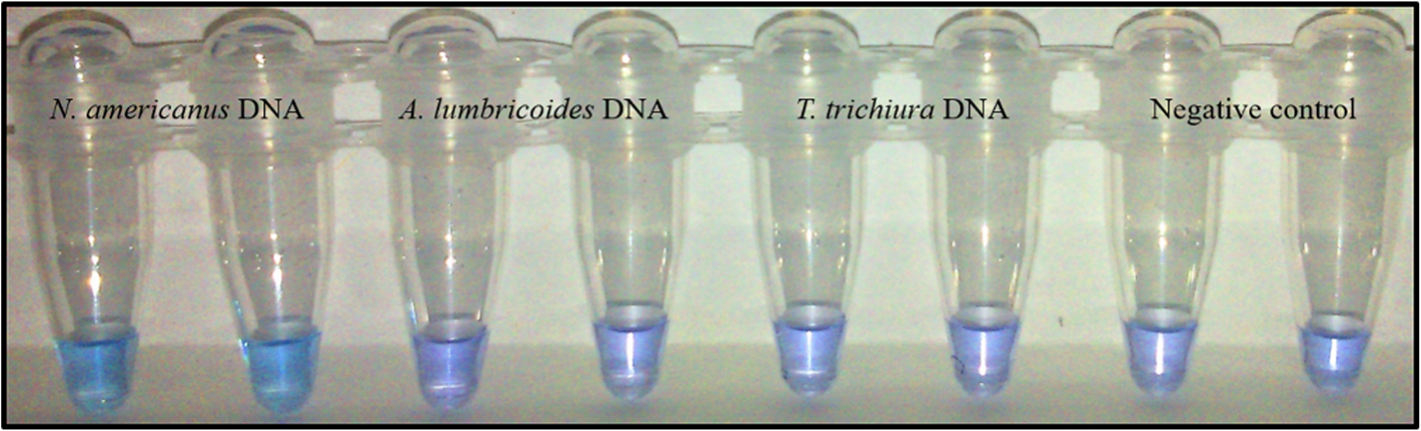
Colourimetric assay for detection of STHs using hydroxy napthol blue (HNB). Colourimetric results of SmartAmp2 assay using *N. americanus* primer set on genomic DNA from *N. americanus* (positive, sky blue), *A. lumbricoides*, *T. trichiura* and negative control (negative, violet)

White precipitate in positive reactions can be detected by eye after brief centrifugation. This precipitate resulted from accumulation of the by-product, magnesium pyrophosphate, whereas negative reactions remained clear. Additionally, results were visually detected by adding the SYBR Green I post-reaction. Negative controls and the non-target DNA remained orange, whereas the positive tubes containing target DNA changed from orange to yellow-green (Fig. 5). No cross amplification was detected. SYBR-green addition post-reaction could result in the introduction of contaminants and the possibility of false positive results. As an alternative, the SmartAmp2 reaction products were visually inspected by adding HNB dye to the reaction mixture. The pre-addition of HNB to reaction master mix did not inhibit amplification efficiency. The colour changes from violet (negative reaction) to sky blue (positive reaction) with positive reactions (Fig. 6). Assays can be run with species-specific primer sets in different tubes to detect the presence or not of each species.

### Validation of STH diagnostic assays on field samples

Positive field samples [*A. lumbricoides* (*n* = 21), *N. americanus* (*n* = 19), and *T. trichiura* (*n* = 15)] were tested in SmartAmp2 assays. Pools of eggs (*A. lumbricoides* and *T. trichiura*) or pools of larvae (*N. americanus*) previously collected under microscopy for each stool sample were analyzed. SmartAmp2 amplification was detected in all the samples within 30–40 min. No amplification was observed from negative controls. Positive controls were always included. Similarly, assays were tested on DNA from single eggs and the results showed that the real-time detection of single egg DNA could be performed in 40–50 min. The minimal detection time for a single egg corresponded to that of approximately 10 pg of adult genomic DNA.

### Evaluation of SmartAmp2 assay in spiked faecal samples

**Fig. 7:**
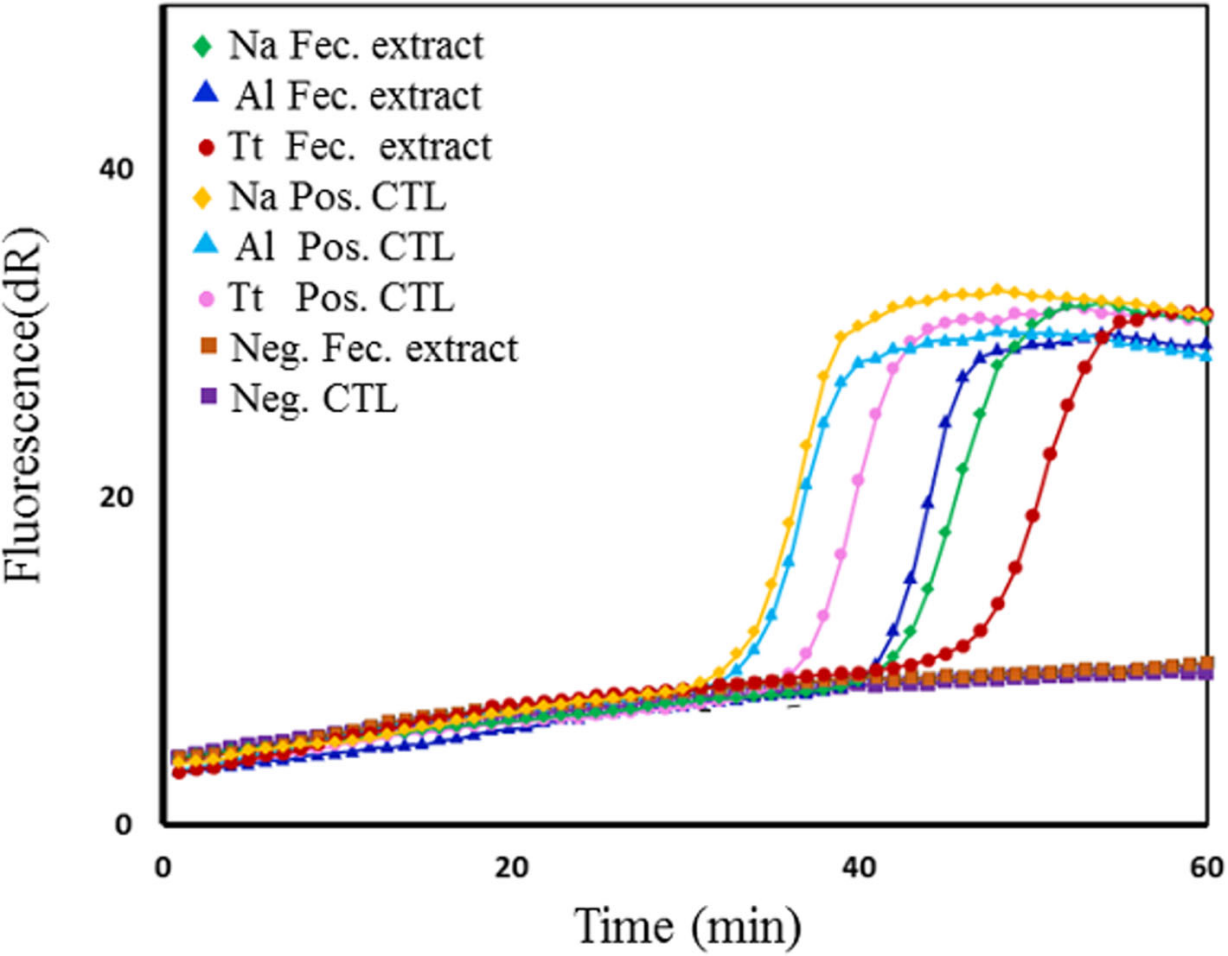
Evaluation of *Bst* polymerase tolerance to inhibitors in spiked faecal samples*.* SmartAmp2 assay amplifications using faecal samples (Fec.) spiked with *N. americanus* (Na) larvae, *A. lumbricoides* (Al) eggs and *T. trichiura* (Tt) eggs. *Abbreviations*: Fec. extract, faecal extract; Pos. CTL, positive controls; Neg. Fec. Extract, non-spiked negative faecal extract; Neg. CTL, negative control

To further assess the performance of the SmartAmp2 assay, spiked-faecal samples with eggs/larvae and non-spiked (negative) faecal samples were assessed in triplicate in the SmartAmp2 assay. DNA was extracted using simple NaOH treatment and a heating step, which was sufficient to release the DNA from eggs/larvae. High amplification efficiency was achieved when crude faecal extracts were diluted 4-fold. In addition, the assay sensitivity improved by adding BSA to the reaction mixture. BSA did not interfere with the colour change of HNB. Real-time amplification was obtained within 40 min only from positive (spiked) faecal extract, using a species-specific primer set, whereas the non-spiked faecal extract and the negative controls (dH_2_O) remained at base line for at least 90 min (Fig. 7). This indicates the high specificity of the assay and the high tolerance of the *Bst* polymerase to inhibitors even when crude sample preparations were used. The presence of faecal matter caused a few minutes’ delay compared with the positive control (without faecal matter).

## Discussion

We have developed rapid and sensitive isothermal diagnostic assays based on the SmartAmp2 method for detection of *T. trichiura*, *A. lumbricoides* and *N. americanus.* The assay was optimized to detect STH DNA in faecal extracts. Optimized species-specific primer sets rapidly amplified a specific target region in the β-tubulin gene with no cross amplification from non-target DNA. Additionally, the SmartAmp2 assay achieved high specificity for detecting a specific target sequence in individual and mixed STH DNAs with no cross amplification from DNA of closely related species.

For species detection, target DNAs should vary enough in sequence to allow the differentiation of species, but exhibit no, or minor, variation within a species [40]. Previous studies have shown that the first internal transcribed spacer (ITS1) and second internal transcribed spacer (ITS2) of nuclear ribosomal DNA represent reliable genetic markers for the identification of a wide range of parasites [40, 41] due to high inter-species and low intra-species variation (low mutation rate). However, the sensitivity of a molecular assay could be compromised by variation in the target gene sequence across different geographical regions (intra-species variation). In *N. americanus*, high levels of genetic variation in mitochondrial and ribosomal DNA have been identified, suggesting distinct genotypes [40]. In this study, the highly conserved single-copy β-tubulin gene was used as a target for diagnosis of STHs. Based on our previous experience in developing SNP genotyping assays using this method [42, 43], we found that SmartAmp2 has the capability to detect a target sequence in high homology regions, with no cross amplification of closely related genes or species. In fact, targeting a highly conserved gene provides the advantage that there is likely to be little or no variation within a species when samples are obtained in different parts of the world. This ensures the high efficiency of the assay particularly with a highly sensitive method such as SmartAmp2.

Faeces may contain a wide variety of living organisms. Isolated faecal genomic DNA may challenge the sensitivity and specificity of a molecular diagnostic test. In this study, a basic local alignment search tool (BLAST) search of GenBank was performed to identify cross-reactivity in silico, but assays were not tested (in vitro) on DNA from other intestinal parasites such as *S. stercoralis* that could co-infect individuals. Theoretically, the SmartAmp2 primers should not amplify non-target DNA as the assay is highly specific, using four species-specific primers with asymmetrical design that prevents alternative misamplification pathways. The ability of the assay to distinguish the STHs based on a highly conserved gene, such as β-tubulin, indicates the high specificity of the assay in detection of STHs in faecal samples. However, further clinical validation on control DNA samples from other intestinal parasites would support these findings. Our *N. americanus*-specific primer set was designed to detect *N. americanus* DNA but not *A. duodenale* DNA; however, the *A. duodenale* assay was not tested as *A. duodenale* DNA was not available. A limitation of LAMP assays is that they cannot so far be multiplexed, so that separate assays in a multiwall plate with the species-specific primers, need to be run for each STH species of interest.

STH SmartAmp2 assays allowed detection of as little as 1 pg genomic DNA within 50–60 min and the detection of single egg DNA within 40–50 min. However, further work needs to be done to compare the results quantitatively with traditional egg count methods, such as the Kato Katz assay. The asymmetrical primer design, using four specific primers that target five distinct sequences on the target DNA all successfully contributed to the high sensitivity of the assay, particularly when targeting a single copy gene such as the β-tubulin gene. Additionally, the *Bst* 2.0 DNA polymerase showed improved speed and yield compared with the wild-type *Bst* DNA polymerase Large Fragment and also showed no difference from the *Aac* polymerase used in SmartAmp2 [39].

The colourimetric STH assay using HNB, a metal indicator for calcium and a reagent for alkaline earth metal ions [29], allowed the detection of STH DNAs without inhibition of the amplification efficiency. A positive reaction was indicated by a colour change from violet to sky blue. This HNB assay could also be conducted in a 96-well microplate. Furthermore, the pre-addition of the HNB to the reaction mix is one of the important advantages that keeps the assay in a closed system and eliminates the need for post-reaction manipulation, thus reducing the risk of cross-contamination. Other significant advantages to the use of HNB are low cost, read-out by eye and stability at room temperature [44]. HNB does not inhibit amplification and provides a detection threshold similar to SYBR Green [29]. One possible limitation is the sensitivity of HNB to pH changes, calcium and magnesium ion concentrations. The assay conditions should be standardized with optimal conditions to maintain the reliability of assay and the interpretation of the colour change.

Faecal samples spiked with STH eggs and processed with the SmartAmp2 assay showed high specificity of the assay and high tolerance of *Bst* polymerase to faecal inhibitors in crude sample preparations. The pre-addition of BSA to the reaction mixture improved assay sensitivity and amplification efficiency. A simple and modified DNA extraction method with NaOH (alkaline treatment) was used to liberate the nucleic acid from the eggs/larvae. Alkaline treatment is a simple, inexpensive and efficient extraction method to release DNA for PCR amplification and has been applied in previous studies [45–47]. Alkaline treatment with NaOH has been used for DNA extraction from blood and tissue for detection of cancer cell associated genotypes using the SmartAmp2 method [35]. The *Bst* polymerase is highly tolerant of inhibitors in clinical samples [39]. However, faecal material is a complex matrix and DNA isolation from faecal samples is usually combined with substances that could inhibit the amplification or compromise the assay sensitivity. Further work is needed to examine the assay sensitivity using different DNA extraction methods and to compare crude DNA preparations, obtained from simple alkaline treatment (NaOH) protocols, with commercial DNA extraction methods.

In this study, we developed a new diagnostic assay to detect three of the most important STHs, but the assay is relatively flexible and can be adjusted and adapted for the identification of new targets and hence its application can be expanded in resource-limited settings. The colourimetric STH assay is cost-effective. Primers used in this study were not HPLC-purified, and in-house prepared buffers and reagents were used. The main expense would be the DNA extraction, which depends on whether a commercial DNA extraction protocol is used or a simple, inexpensive alkaline DNA extraction method.

The colourimetric STH assay, like other isothermal (LAMP) assays, is not fully quantitative but proved to be rapid, simple and robust with the potential to be adapted in field conditions. Because the response time is sensitive to the amount of species-specific DNA, the assay can be semi-quantitative and be used to monitor response to treatment and to estimate approximate drug efficacy. However, the assay still requires storage of the reagents at -20 °C. The requirement for a cold chain could be overcome by developing a dry-reagent based assay using lyophilized reagents, as previously used in other LAMP assays [48, 49].

## Conclusions

Novel STH diagnostic assays based on the SmartAmp2 method were developed and applied to field samples. The colourimetric detection assays proved to be rapid, sensitive and highly specific with the potential to be applied in resource-constrained settings. However, further work is needed to validate the assays on a larger number of faecal samples.

## Additional file

Additional file 1: Figure S1. Comparative alignment of a specific target region in the β-tubulin isotype 1 gene. (DOCX 62 kb)

## Abbreviations

BP: Boost primer
FP: Folding primer
HNB: Hydroxy napthol blue
KK: Kato Katz
LAMP: Loop-mediated isothermal amplification
MDA: Mass drug administration
OP: Outer primer
STH: Soil-transmitted helminths
TP: Turn-back primer

## References

[1] Bethony J, Brooker S, Albonico M, Geiger SM, Loukas A, Diemert D (2006). Soil-transmitted helminth infections: ascariasis, trichuriasis, and hookworm. Lancet.

[2] Prichard RK, Basanez MG, Boatin BA, McCarthy JS, Garcia HH, Yang GJ (2012). A research agenda for helminth diseases of humans: intervention for control and elimination. PLoS Negl Trop Dis.

[3] Colley DG, LoVerde PT, Savioli L (2001). Infectious disease. Medical helminthology in the 21st century. Science.

[4] McCarty TR, Turkeltaub JA, Hotez PJ (2014). Global progress towards eliminating gastrointestinal helminth infections. Curr Opin Gastroenterol.

[5] WHO (2011). Helminth control in school age children: a guide for managers of control programmes.

[6] WHO. Soil-transmitted Helminthiases: STH: Eliminating Soil-transmitted Helminthiases as a Public Health Problem in Children: Progress Report 2001–2010 and Strategic Plan 2011-2020. Geneva: World Health Organization; 2012.

[7] Albonico M, Levecke B, LoVerde PT, Montresor A, Prichard R, Vercruysse J (2015). Monitoring the efficacy of drugs for neglected tropical diseases controlled by preventive chemotherapy. J Glob Antimicrob Resist.

[8] Montresor A, AP N, Albonico M, Gabrielli AF, Jankovic D, Fitzpatrick C (2015). Soil-transmitted helminthiasis: the relationship between prevalence and classes of intensity of infection. Trans R Soc Trop Med Hyg.

[9] Harhay MO, Horton J, Olliaro PL, Utzinger J (2011). Diagnostics are central for a truly holistic approach against intestinal parasitic diseases. Int J Infect Dis.

[10] McCarthy JS, Lustigman S, Yang GJ, Barakat RM, Garcia HH, Sripa B (2012). A research agenda for helminth diseases of humans: diagnostics for control and elimination programmes. PLoS Negl Trop Dis.

[11] Katz N, Chaves A, Pellegrino JA (1972). Simple device for quantitative stool thick-smear technique in schistosomiasis mansoni. Rev Inst Med Trop Sao Paulo.

[12] Knopp S, Mgeni AF, Khamis IS, Steinmann P, Stothard JR, Rollinson D (2008). Diagnosis of soil-transmitted helminths in the era of preventive chemotherapy: effect of multiple stool sampling and use of different diagnostic techniques. PLoS Negl Trop Dis.

[13] Pullan RL, Smith JL, Jasrasaria R, Brooker SJ (2014). Global numbers of infection and disease burden of soil transmitted helminth infections in 2010. Parasit Vectors.

[14] Dacombe RJ, Crampin AC, Floyd S, Randall A, Ndhlovu R, Bickle Q (2007). Time delays between patient and laboratory selectively affect accuracy of helminth diagnosis. Trans R Soc Trop Med Hyg.

[15] Krolewiecki AJ, Ramanathan R, Fink V, McAuliffe I, Cajal SP, Won K (2010). Improved diagnosis of *Strongyloides stercoralis* using recombinant antigen-based serologies in a community-wide study in northern Argentina. Clin Vaccine Immunol.

[16] Petri WA (2000). Protozoan parasites that infect the gastrointestinal tract. Curr Opin Gastroenterol.

[17] Basuni M, Muhi J, Othman N, Verweij JJ, Ahmad M, Miswan N (2011). A pentaplex real-time polymerase chain reaction assay for detection of four species of soil-transmitted helminths. Am J Trop Med Hyg.

[18] Becker SL, Piraisoody N, Kramme S, Marti H, Silue KD, Panning M (2015). Real-time PCR for detection of *Strongyloides stercoralis* in human stool samples from cote d'Ivoire: diagnostic accuracy, inter-laboratory comparison and patterns of hookworm co-infection. Acta Trop.

[19] Carlsgart J, Roepstorff A, Nejsum P, Multiplex PCR (2009). On single unembryonated *Ascaris* (roundworm) eggs. Parasitol Res.

[20] Gordon CA, McManus DP, Acosta LP, Olveda RM, Williams GM, Ross AG (2015). Multiplex real-time PCR monitoring of intestinal helminths in humans reveals widespread polyparasitism in Northern Samar, the Philippines. Int J Parasitol.

[21] Ndao M (2009). Diagnosis of parasitic diseases: old and new approaches. Interdiscip Perspect Infect Dis.

[22] Verweij JJ, Pit DS, van Lieshout L, Baeta SM, Dery GD, Gasser RB (2001). Determining the prevalence of *Oesophagostomum bifurcum* and *Necator americanus* infections using specific PCR amplification of DNA from faecal samples. Tropical Med Int Health.

[23] de Gruijter JM, van Lieshout L, Gasser RB, Verweij JJ, Brienen EA, Ziem JB (2005). Polymerase chain reaction-based differential diagnosis of *Ancylostoma duodenale* and *Necator americanus* infections in humans in northern Ghana. Tropical Med Int Health.

[24] Notomi T, Okayama H, Masubuchi H, Yonekawa T, Watanabe K, Amino N (2000). Loop-mediated isothermal amplification of DNA. Nucleic Acids Res.

[25] Mori Y, Kanda H, Notomi T (2013). Loop-mediated isothermal amplification (LAMP): recent progress in research and development. J Infect Chemother.

[26] Tomita N, Mori Y, Kanda H, Notomi T (2008). Loop-mediated isothermal amplification (LAMP) of gene sequences and simple visual detection of products. Nat Protoc.

[27] Goto M, Honda E, Ogura A, Nomoto A, Hanaki K (2009). Colorimetric detection of loop-mediated isothermal amplification reaction by using hydroxy naphthol blue. BioTechniques.

[28] Nie K, Zhao X, Ding X, Li XD, Zou SM, Guo JF (2013). Visual detection of human infection with influenza a (H7N9) virus by subtype-specific reverse transcription loop-mediated isothermal amplification with hydroxynaphthol blue dye. Clin Microbiol Infect.

[29] Poole CB, Tanner NA, Zhang Y, Evans TC, Carlow CK (2012). Diagnosis of brugian filariasis by loop-mediated isothermal amplification. PLoS Negl Trop Dis.

[30] Drame PM, Fink DL, Kamgno J, Herrick JA, Nutman TB (2014). Loop-mediated isothermal amplification for rapid and semiquantitative detection of *Loa loa* infection. J Clin Microbiol.

[31] Fernandez-Soto P, Sanchez-Hernandez A, Gandasegui J, Bajo Santos C, Lopez-Aban J, Saugar JM (2016). Strong-LAMP: a LAMP assay for *Strongyloides* spp. detection in stool and urine samples. Towards the diagnosis of human strongyloidiasis starting from a rodent model. PLoS Negl Trop Dis.

[32] Watts MR, James G, Sultana Y, Ginn AN, Outhred AC, Kong F (2014). A loop-mediated isothermal amplification (LAMP) assay for *Strongyloides stercoralis* in stool that uses a visual detection method with SYTO-82 fluorescent dye. Am J Trop Med Hyg..

[33] Mugambi RM, Agola EL, Mwangi IN, Kinyua J, Shiraho EA, Mkoji GM (2015). Development and evaluation of a loop mediated isothermal amplification (LAMP) technique for the detection of hookworm (*Necator americanus*) infection in fecal samples. Parasit Vectors.

[34] Shiraho EA, Eric AL, Mwangi IN, Maina GM, Kinuthia JM, Mutuku MW, Mugambi RM, Mwandi JM, Mkoji GM (2016). Development of a loop mediated isothermal amplification for diagnosis of *Ascaris lumbricoides* in fecal samples. J Parasitol Res.

[35] Mitani Y, Lezhava A, Sakurai A, Horikawa A, Nagakura M, Hayashizaki Y (2009). Rapid and cost-effective SNP detection method: application of SmartAmp2 to pharmacogenomics research. Pharmacogenomics.

[36] Diawara A, Halpenny CM, Churcher TS, Mwandawiro C, Kihara J, Kaplan RM (2013). Association between response to albendazole treatment and beta-tubulin genotype frequencies in soil-transmitted helminths. PLoS Negl Trop Dis.

[37] Diawara A, Schwenkenbecher JM, Kaplan RM, Prichard RK (2013). Molecular and biological diagnostic tests for monitoring benzimidazole resistance in human soil-transmitted helminths. Am J Trop Med Hyg..

[38] Lake SL, Matthews JB, Kaplan RM, Hodgkinson JE (2009). Determination of genomic DNA sequences for beta-tubulin isotype 1 from multiple species of cyathostomin and detection of resistance alleles in third-stage larvae from horses with naturally acquired infections. Parasit Vectors.

[39] Hoshi K, Takakura H, Mitani Y, Tatsumi K, Momiyama N, Ichikawa Y (2007). Rapid detection of epidermal growth factor receptor mutations in lung cancer by the SMart-amplification process. Clin Cancer Res.

[40] Gasser RB (2006). Molecular tools - advances, opportunities and prospects. Vet Parasitol.

[41] Gasser RB, Cantacessi C, Campbell BE (2009). Improved molecular diagnostic tools for human hookworms. Expert Rev Mol Diagn.

[42] Rashwan N, Bourguinat C, Keller K, Gunawardena NK, de Silva N, Prichard R (2016). Isothermal diagnostic assays for monitoring single nucleotide polymorphisms in *Necator americanus* associated with benzimidazole drug resistance. PLoS Negl Trop Dis.

[43] Rashwan N, Scott M, Prichard R (2017). Rapid genotyping of beta-tubulin polymorphisms in *Trichuris trichiura* and *Ascaris lumbricoides*. PLoS Negl Trop Dis.

[44] Britton S, Cheng Q, Sutherland CJ, McCarthy JS (2015). A simple, high-throughput, colourimetric, field applicable loop-mediated isothermal amplification (HtLAMP) assay for malaria elimination. Malaria J.

[45] Wang H, Qi M, Cutler AJ (1993). A Simple method of preparing plant samples for PCR. Nucleic Acids Res.

[46] Xin Z, Velten JP, Oliver MJ, Burke JJ, High-throughput DNA (2003). Extraction method suitable for PCR. BioTechniques.

[47] Beltran S, Galinier R, Allienne JF, Boissier J (2008). Cheap, rapid and efficient DNA extraction method to perform multilocus microsatellite genotyping on all *Schistosoma mansoni* stages. Mem Inst Oswaldo Cruz.

[48] Beissner M, Phillips RO, Battke F, Bauer M, Badziklou K, Sarfo FS (2015). Loop-mediated isothermal amplification for laboratory confirmation of Buruli ulcer disease - towards a point-of-care test. PLoS Negl Trop Dis.

[49] Chen HW, Weissenberger G, Ching WM (2016). Development of lyophilized loop-mediated isothermal amplification reagents for the detection of *Leptospira*. Military Med.

[50] Krause RJ, Koski KG, Pons E, Sandoval N, Sinisterra O, Scott ME (2015). *Ascaris* and hookworm transmission in preschool children from rural Panama: role of yard environment, soil eggs/larvae and hygiene and play behaviours. Parasitology.

[51] Krause RJ, Koski KG, Pons E, Sinisterra O, Scott ME (2016). *Ascaris* and hookworm transmission in preschool children in rural Panama: role of subsistence agricultural activities. Parasitology.

