# Supplementary material for: Isothermal diagnostic assays for the detection of soil-transmitted helminths based on the SmartAmp2 method
Source: Parasit Vectors. 2017 Oct 18;10:496. doi: 10.1186/s13071-017-2420-1 (PMC5648480; doi:10.1186/s13071-017-2420-1)
Supplement: Additional file 1: Figure S1. — Comparative alignment of a specific target region in the β-tubulin isotype 1 gene. (DOCX 62 kb) [file 13071_2017_2420_MOESM1_ESM.docx]

**Comparative alignment of a specific target region in the β-tubulin isotype 1 gene.**

**
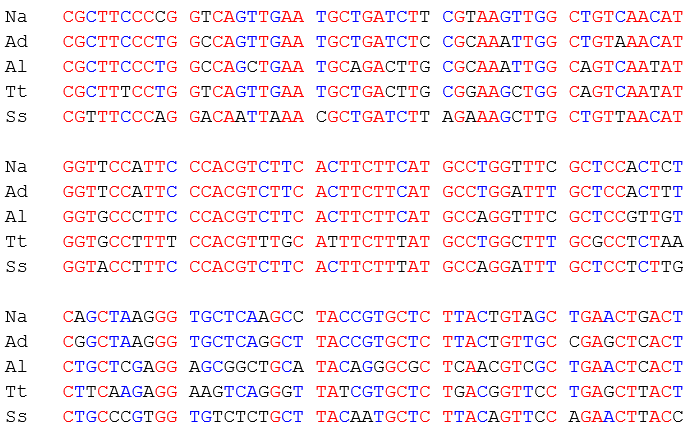
**

**Additional file 1: Fig. S1.** Species-specific SmartAmp2 primer sets were designed based on the sequence divergence between *N. americanus* (Na), *A. duodenale* (Ad)*, A. lumbricoides* (Al), *T. trichiura* (Tt) and *S. stercoralis* (Ss).
